# Small ubiquitin-like modifier-tag and modified protein purification significantly increase the quality and quantity of recombinant African swine fever virus p30 protein

**DOI:** 10.14202/vetworld.2024.1157-1167

**Published:** 2024-05-22

**Authors:** Jullada Chootip, Payuda Hansoongnern, Nattarat Thangthamniyom, Sirin Theerawatanasirikul, Penpitcha Chankeeree, Challika Kaewborisuth, Porntippa Lekcharoensuk

**Affiliations:** 1 Department of Microbiology and Immunology, Faculty of Veterinary Medicine, Kasetsart University, Bangkok, 10900, Thailand; 2 Virology and Cell Technology Research Team, National Center for Genetic Engineering and Biotechnology (BIOTEC), National Science and Technology Development Agency, Pathumthani, 12120, Thailand; 3 Department of Anatomy, Faculty of Veterinary Medicine, Kasetsart University, Bangkok, 10900, Thailand; 4 Center for Advanced Studies in Agriculture and Food, KU Institute of Advanced Studies, Kasetsart University, Bangkok, 10900, Thailand

**Keywords:** African swine fever, diagnosis, indirect enzyme-linked immunosorbent assay, p30, protein expression, purification

## Abstract

**Background and Aim:**

African swine fever (ASF) is a highly virulent and contagious viral disease caused by the ASF virus (ASFV). It has a significant impact on swine production throughout the world, while existing vaccines and specific treatments remain ineffective. ASFV p30 is a potent antigenic protein that induces protective antibodies immediately after infection; however, most recombinant p30 is insoluble. This study aimed to improve the solubility, yield, and purity of recombinant p30 by tagging it with a small ubiquitin-like modifier (SUMO) and modifying the protein purification process.

**Materials and Methods:**

SUMO fused with ASFV p30 (SUMO-p30) and p30 alone were cloned and expressed in *Escherichia coli*. SUMO-p30 and p30 solubility and expression levels were analyzed by sodium dodecyl sulfate-polyacrylamide gel electrophoresis (SDS-PAGE). Protein purification was modified by combining ammonium sulfate precipitation method with affinity chromatography. In addition, large-scale production of all versions of p30 were compared using SDS-PAGE and western blotting, and the purified p30 was used to develop the indirect enzyme-linked immunosorbent assay (ELISA).

**Results:**

The solubility and expression levels of SUMO-p30 were dramatically enhanced compared with that of p30. Modification of the purification process significantly increased purified and soluble SUMO-p30 and p30 yields by 6.59 and 1.02 μg/mL, respectively. Large-scale production confirmed that this procedure increased the quantity of recombinant p30 while maintaining protein purity and immunogenicity. The p30-based indirect ELISA was able to discriminate between positive and negative serum samples with statistically significant differences in mean optical density 450 values (p < 0.001).

**Conclusion:**

This study demonstrates the enhancement of solubility, purity, and yield of ASFV p30 expressed in *E.coli* by SUMO fusion tagging and combining ammonium sulfate precipitation with affinity chromatography for protein purification. These positive effects were sustained in large-scale production. Cleavage and removal of hexahistidine-SUMO tag from the fusion protein by protease may not be suitable when handling a large amount of the protein. However, the SUMO-fused p30 retained strong immunoreactivity to convalescent swine serum, indicating its application in immunization and diagnostic purposes. The expression and purification procedures in this study could be applied to increase solubility, quality, and quantity of other recombinant proteins as well.

## Introduction

African swine fever (ASF) is a viral hemorrhagic disease affecting domestic pigs as well as wild boars, resulting in high mortality and morbidity rates. Historical records trace its first appearance in Kenyan wild boar in 1921, where it was transmitted by a soft tick in the sylvatic cycle [1]. In the same year, a highly virulent and contagious ASF with a mortality rate of 100% emerged among domestic pigs and became widespread across Africa, subsequently extending its reach to Europe, Russia, Brazil, and the Caribbean before being eradicated in the mid-19^th^ century [2]. However, a recent pan-epizootic ASF erupted in Georgia in 2007, rapidly spreading across the country and into the European Union, Russia, and Asia [3]. Following the first ASF outbreak in China, the world’s largest pork producer and consumer [4], the disease has now been distributed to 16 Asian countries with confirmed ASF cases in pigs [5]. The panzootic nature of ASF has had a profound negative impact on global economics and food security [6], and it is important to note that no commercial vaccines or specific treatments for ASF are currently available. The ASF virus (ASFV) belongs to the *Asfarviridae* family, representing a double-stranded DNA arbovirus with complex enveloped proteins [7]. Viral particles comprise more than 100 different proteins, each with functions related to viral structure, viral entry, morphogenesis, transcription, RNA modification, maintenance of genome integrity, and evasion of host defense mechanisms [8]. Among these, 14 viral proteins, including p30, p54, p72, A104R, p10, F334L, K196R, NP419L, B602L, C44L, Cp312R, and K205R, have been identified as antigenic, containing the immunogenic determinants essential for developing a serological diagnostic tool [9]. p30 is a phosphorylated 30 kDa protein of ASFV located in the inner envelope layer of the virion [9]. It is encoded by *the CL204P* gene and is expressed from early to late stages of infection. The function of p30 is related to viral attachment and internalization. In addition, p30 is highly immunogenic and induces the production of ASFV-specific neutralizing antibodies [10]. This property has resulted in its use in various diagnostic assays, such as immunoblotting [11], dual matrix indirect enzyme-linked immunosorbent assay (ELISA) [12], and blocking ELISA based on p30-specific monoclonal antibody [13].

*Escherichia coli* is commonly used for producing recombinant proteins in prokaryotic expression systems due to its ability to generate high protein yield with low production costs. Nevertheless, this system has drawbacks, such as a lack of post-translational modification and frequent accumulation of insoluble proteins known as inclusion bodies [14], causing protein misfolding and insolubility, respectively. Several solutions have been developed to address these challenges in *E. coli* protein expression. One promising method involves tagging protein of interest with a small ubiquitin-like modifier (SUMO). SUMO is a conserved 10-kDa polypeptide that functions in the post-translational modification of eukaryotic cellular proteins [15]. When the SUMO protein is fused in-frame with the protein of interest, it can enhance the expression level and improve the solubility of the fusion protein [16]. Furthermore, tag cleavage is very convenient because SUMO protease is highly specific and recognizes the tertiary structure of the cleavage site rather than the linear amino acid sequence [17]. Importantly, SUMO protease does not cleave the protein of interest [18]. SUMO-tag has been used in conjunction with the hexahistidine tag to facilitate the purification process [19–25].

The primary objective of this study was to enhance the expression level and improve the solubility of the p30 protein within the prokaryotic system using SUMO fusion tag technology. In addition, the presence of contaminant proteins can be reduced by salting with ammonium sulfate before affinity chromatography purification. In addition, this study demonstrates the specific reactivity between p30 and convalescent swine serum and the potential use of p30 for ASF detection.

## Materials and Methods

### Ethical approval

This study did not involve live animals. All serum samples used in this study were obtained after the diagnostic process had been completed.

### Study period and location

This study was conducted from May 2020 to November 2023 at Faculty of Veterinary Medicine, Kasetsart University, and National Center for Genetic Engineering and Biotechnology (BIOTEC).

### Construction of recombinant expression vectors

*The CP204L* gene encoding p30 protein (GenBank accession number: OR567419) cloned in pUC57 (Genescript, Piscataway, NJ, USA) was used as a template to amplify the *CP204L* gene with a pair of specific primers: forward primer; 5’TAGCATGCATGGCATCAGGAGGAGC- 3’ (containing a *Sph*I restriction site) and reverse primer; 5’-TAGTCGACTTAGGTACTGTAACGCAG -3’ (containing a *Sal*I restriction site) using Phusion™ high-fidelity DNA polymerase (Thermo Fisher Scientific, Waltham, Massachusetts, USA). Polymerase chain reaction (PCR) conditions consisted of initial denaturation at 98°C for 30 s, followed by 35 cycles of denaturation at 98°C for 10 s, annealing at 60°C for 15 s, extension at 72°C for 1 min, and final extension at 72°C for 10 min. The PCR product was purified and subsequently cloned into pQE-80L (Qiagen, Hilden, Germany) at the *Sph*I and *Sal*I sites to produce a recombinant vector expressing the p30 protein fused with a histidine tag (6His tag) at the N-terminus, referred to as pQE_p30. The same PCR product was also cloned into pSMst (an in-house plasmid) that included 6His and yeast SUMO of *Saccharomyces cerevisiae* (*SMT3* gene) located upstream of the multiple cloning sites. This process generates a recombinant vector expressing the p30 protein fused with 6His and SUMO tags at the N-terminus, designated as pSMst_p30. The p30 nucleotide sequence was verified using DNA sequencing (Macrogen, Korea).

### Protein expression and identification of protein expression form

Protein expression was performed as previously described by Sariya *et al*. [26]. In brief, plasmids pQE_p30 and pSMst_p30 were individually transformed into the *E. coli* strain BL21. *E. coli* cells containing the recombinant plasmids were cultured in Luria-Bertani (LB) broth containing 100 μg/mL of ampicillin and incubated at 37°C with orbital shaking at 225 rpm for 16 h. Subsequently, 1 mL of the overnight bacterial culture was transferred to a flask containing 100 mL of LB broth containing ampicillin and incubated at 37°C with shaking at 225 rpm. Isopropyl β-D-thiogalactoside (Sigma-Aldrich, St. Louise, MI, USA) was added to the bacterial culture at a final concentration of 0.5 mM when the optical density at 600 nm (OD600) of the cultures reached 0.4–0.6 to induce protein expression. Subsequently, the incubation was continued at 20°C with shaking at 225 rpm for 20 h.

Bacterial cells were harvested by centrifugation at 2851 × *g* at 4°C for 15 min. Subsequently, the culture medium was removed, and the bacterial cell pellets were resuspended in lysis buffer (50 mM NaH_2_PO_4_, pH 8.0, 300 mM NaCl, 1% Nonidet P-40, 1 mg/mL lysozyme, 1 mM phenylmethylsulfonyl fluoride [PMSF], 1 μg/mL leupeptin, 1 μg/mL aprotinin, and 1 μg/mL pepstatin A). Subsequently, the suspension was incubated on ice for 1 h, followed by sonication with an output of 30% for 5 min using Sonics Vibra-Cell™ ultrasonic processor with a ½-inch (13 mm) probe (Sonics & Materials, Inc., Connecticut, USA). The cell lysates were then centrifuged at 7825 × *g* at 4°C for 15 min. The supernatant containing native proteins was collected as a soluble fraction. The remaining pellets were resuspended in denaturing buffer (50 mM NaH_2_PO_4_, pH 8.0, 300 mM NaCl, and 8 M urea). Subsequently, the suspension was incubated on ice for 1 h and spun at 7825 × *g* at 4°C for 15 min. Supernatants containing denatured proteins were collected and retained as an insoluble fraction. Protein samples were analyzed by 10% sodium dodecyl sulfate-polyacrylamide gel electrophoresis (SDS-PAGE) to determine the quality of the expressed protein.

### Partial purification of p30 and SUMO-p30 proteins using ammonium sulfate

After collecting the soluble p30 protein, a significant amount of contamination was observed, which may interfere with the subsequent affinity chromatography purification process. In this study, ammonium sulfate precipitation protocol was optimized according to the method described by Burgess [27] to determine the ideal ammonium sulfate saturation percentage for the partial purification of p30 and SUMO-p30 with some modifications. In brief, ammonium sulfate was added to 10 mL of protein solution to achieve saturation levels of 10%, 20%, 30%, 40%, 50%, and 60%. The protein and ammonium sulfate mixtures were gently stirred and incubated on ice for 60 min. Precipitated proteins were collected by centrifugation at 7825 × *g* at 4°C for 20 min. The obtained pellet was resuspended in 500 μL phosphate-buffered saline (PBS) (Sigma, St. Louis, Missouri, USA) and subjected to SDS-PAGE using 10% gel for protein profile analysis. As soon as appropriate ammonium sulfate saturation percentages were obtained for protein precipitation, a large-scale partial purification of p30 and SUMO-p30 proteins was performed. In the pre-clear step, saturated ammonium sulfate was first slowly added to the p30 and SUMO-p30 cell lysates at final concentrations of 10% and 30%, respectively. These mixtures were gently stirred on ice for 1 h, followed by centrifugation at 7825 × *g* at 4°C for 30 min. Supernatants containing p30 or SUMO-p30 were collected for the next step, and the contaminated proteins in the pellet were discarded. In the second precipitation step, ammonium sulfate was added to the collected supernatants to obtain final concentrations of 20% and 60% for p30 and SUMO-p30, respectively. Protein solutions were gently stirred while incubating on ice for 1 h and then centrifuged at 7825 × *g* at 4°C for 30 min. The supernatant was removed, and the precipitated p30 and SUMO-p30 were resuspended in 10 mL of binding buffer (50 mM NaH_2_PO_4_, pH 8.0, 300 mM NaCl, and 10 mM imidazole) for the affinity chromatography step. To eliminate ammonium salts, the resuspended solutions were subsequently desalted by passing through a PD-10 desalting column (GE Healthcare, Chicago, Illinois, USA), following the manufacturer’s guidelines.

### Purification of p30 and SUMO-p30 proteins using affinity chromatography

The desalted protein solution was further purified by immobilized metal ion affinity chromatography using Protino^®^ Ni-iminodiacetic acid (IDA) resin (Macherey-Nagel, Düren, Nordrhein-Westfalen, Germany) following the manufacturer’s recommended procedure. Briefly, the column was initially equilibrated with an equilibration buffer containing 50 mM NaH_2_PO_4_ (pH 8.0) and 300 mM NaCl. Subsequently, the protein solution from the previous step was applied to the column, and a copious amount of the binding buffer (50 mM NaH_2_PO_4_, pH 8.0, 300 mM NaCl, and 10 mM imidazole) was introduced to remove the unbound proteins away. The target protein was eluted using an elution buffer containing 50 mM NaH_2_PO_4_ (pH 8.0), 300 mM NaCl, and 100 mM imidazole. Each protein fraction was examined by SDS-PAGE using 12% polyacrylamide gels (Bio-Rad Laboratories, Hercules, California, USA). The eluted protein fractions containing each protein were pooled and concentrated using Amicon^®^ ultracentrifugal filters with a pore size of 10-kDa (Millipore, Burlington, Massachusetts, USA). Subsequently, the purified protein concentration was measured using Pierce™ 660 nm Protein Assay (Thermo Fisher Scientific, Waltham, Massachusetts, USA), according to the manufacturer’s instructions. The protein yield was calculated as micrograms per mL.

### Cleavage and removal of SUMO-tag

SUMO protease 1 (LifeSensors, Malvern, Pennsylvania, USA) was used to cleave 100 mg of SUMO-p-30 protein. Enzyme was mixed with the protein solution at a final concentration of 10 units/μL in a total volume of 1500 μL. The reaction tube was incubated at 30°C for 1 h. Following incubation, the cleaved 6His-SUMO-tag was removed by reintroducing the sample into the Ni-IDA resin column. Without the 6His-SUMO-tag, the p30 protein was unable to bind to the resin and passed through the column in the flow-through fraction. The 6His-SUMO-tag bound to the resin was eluted using an elution buffer containing 250 mM imidazole. Each protein fraction was analyzed by SDS-PAGE using 12% polyacrylamide gel (Bio-Rad Laboratories). SUMO-cleaved p30 protein concentration was determined using Pierce™ 660 nm Protein Assay (Thermo Fisher Scientific). The yield of the SUMO-cleaved p30 protein was calculated as micrograms per mL.

### Examination of p30 immunogenicity

To confirm whether the expressed proteins were indeed ASFV p30, western blotting [26] was performed to demonstrate the reactivity between different versions of p30 with an anti-p30 monoclonal antibody and a convalescent swine serum. Five microliters of each recombinant protein, namely, p30, SUMO-p30, SUMO-cleaved p30, and porcine circovirus Type 2 (PCV2) capsid protein [28] (serving as a negative control), were separated using SDS-PAGE with 12% polyacrylamide gels (Bio-Rad Laboratories). Separate protein bands were transferred onto a nitrocellulose membrane (Bio-Rad Laboratories). Membranes were equilibrated in tris-buffered saline tween-20 (TBST) (50 mM Tris-HCl pH 7.5, 150 mM NaCl, and 0.1% v/v Tween-20) and incubated with blocking buffer (50 mM Tris-HCl pH 7.5, 150 mM NaCl, and 3% bovine serum albumin [BSA]) for 1 h at 25°C. The membrane was subsequently incubated with an anti-p30 monoclonal antibody (MyBioSource, San Diego, California, USA) at a dilution of 1:4000 in blocking buffer for 1 h. After three washes with TBST, the membrane was further incubated with anti-mouse immunoglobulin (Ig) G horseradish peroxidase (HRP) (Sigma, St. Louis, Missouri, USA) at a dilution of 1:1000 for 1 h. Finally, the membrane was washed three times with TBST, and the bound antibodies were detected using a 3,3’,5,5’-Tetramethylbenzidine peroxidase substrate kit (Sera Care, Milford, Massachusetts, USA).

Furthermore, reactivity between p30 and a convalescent serum obtained after diagnostic testing was examined by western blotting as described above. Primary and secondary antibodies were ASFV convalescent swine serum at a dilution of 1:200 and anti-pig IgG HRP (Sigma) at a dilution of 1:1000, respectively. The bovine ephemeral fever virus G protein served as a negative control in this procedure [29].

### Large-scale expression and purification of p30 and SUMO-p30

To evaluate the increase in recombinant protein production, *E. coli* strain BL21 expressing p30 and SUMO-p30 proteins were cultured in 2 L under conditions similar to those used for small-scale production. After harvesting, the cell pellet in the lysis buffer (50 mM NaH_2_PO_4_, pH 8.0, 300 mM NaCl, 1% NP-40, 1 mg/mL lysozyme, 1 mM PMSF, 1 μg/mL leupeptin, 1 μg/mL aprotinin, and 1 μg/mL pepstatin A) was resuspended at 5 mL of lysis buffer per 1 g of cell pellet and incubated on ice for an hour. The proteins were extracted by sonication. The cell lysates were centrifuged at 7825 × *g* at 4°C for 15 min, and the supernatant containing the soluble protein was collected for further purification. As regards protein purification, the supernatants obtained from each liter of p30 and SUMO-p30 were purified only by affinity chromatography, whereas the remaining liters were partially purified by ammonium sulfate precipitation and affinity chromatography. The ammonium precipitation procedure was similar to that mentioned earlier, except for the incubation periods of 5 and 16 h in the first and second precipitation steps, respectively. The pellet was resuspended in 100 mL of binding buffer (20 mM NaH_2_PO_4_, pH 8.0, 0.5 M NaCl, and 20 mM imidazole) and then desalted using a PD-10 column (GE Healthcare, Uppsala, Sweden). The affinity chromatography system was replaced by a prepacked HisTrap-HP column (Cytiva, Uppsala, Sweden) using the AKTA™ start chromatography system (GE Healthcare) for convenience during the large-scale protein purification. The filtered sample was loaded into the column. Subsequently, the unbound protein was washed with 10 mL of wash buffer (20 mM NaH_2_PO_4_, pH 8.0, 0.5 M NaCl, and 50 mM imidazole). The target protein was eluted using an elution buffer (50 mM NaH_2_PO_4_, pH 8.0, 300 mM NaCl, and 100–500 mM imidazole). We pooled and concentrated the purified protein fractions and determined the protein concentration, as described above. To compare the efficiency of protein purification methods, 3 μg of purified p30 and SUMO-p30 with and without ammonium precipitation was loaded into each well of 12% polyacrylamide gel (Bio-Rad Laboratories) and analyzed by SDS-PAGE. Furthermore, the immunogenicity of these purified proteins was examined by western blotting as previously described, but with 0.5 μg of protein per well.

### Indirect ELISA based on SUMO-cleaved p30

The p30 indirect ELISA was established using SUMO-cleaved p30, as described elsewhere in this paper [30]. Initially, the SUMO-cleaved p30 concentration was optimized, and pig serum and anti-pig conjugated HRP were diluted using checkerboard titration. All sera used in this study were obtained after the completion of the diagnostic process. SUMO-cleaved p30 was diluted in 0.1 M carbonate buffer at pH 9.5 to final concentrations of 1, 0.5, 0.25, 0.125, 0.065, and 0.031 μg/mL. These concentrations were coated at 100 μL in each well of the 96-well plates and incubated overnight at 4°C. Antigen-coated plates were washed 3 times with phosphate-buffered saline tween-20 (PBST) (PBS pH 7.4, 0.05% v/v Tween-20), followed by incubation with a blocking buffer (PBS pH 7.4, 0.05% v/v Tween-20, 2% BSA) at 25°C for 1 h. The plates were then washed with PBST and incubated with two-fold serially diluted swine serum samples in a diluent buffer (PBS pH 7.4, 0.05% v/v Tween-20, 0.5% BSA) starting at 1:400–1:3200 for 1 h at 37°C. Following serum incubation, the plates were washed three times with PBST and incubated at 37°C for 1 h with anti-pig IgG HRP at a dilution of 1:20,000. Excess antibodies were washed away with PBST, and bound anti-pig IgG HRP was incubated with tetramethylbenzidine solution (Biolegend, San Diego, California, USA) in the dark for 15 min. Finally, the reaction was stopped by adding 100 μL of 2M H_2_SO_4_. The OD450 of the developed color in each well was measured at a wavelength of 450 nm and recorded using a microplate reader (BioTek Synergy H1, Agilent Technologies, Inc, Santa Clara, California).

To set a cutoff for the assay, 50 swine sera, classified as ASF positive and negative by a commercial indirect ELISA kit (ID Screen® ASF Indirect, Innovative Diagnostics, Louis Pasteur, France), were tested in the p30 indirect ELISA. The indirect ELISA cutoff value was determined by calculating the mean OD450 of the negative sera plus three-fold standard deviations (X̅ + 3SD). The p30 indirect ELISA was evaluated using 200 swine sera, including 128 and 72 previously determined as positive and negative samples, respectively, using the ID Screen® Innovative Diagnostics, Grabels, France.

Four positive and four negative serum samples were used in each experiment for reproducibility assays. Five replicates of each serum sample were assigned to the same plate for intra-assay, and five replicates of each sample were assigned to different plates for inter-assay. Means, SD, and coefficients of variation (CVs) were calculated. Cross-reactivity of the recombinant p30 protein with sera specific to porcine reproductive and respiratory syndrome virus (PRRSV), swine influenza virus (SIV), and PCV2 was also evaluated using indirect ELISA.

### Statistical analysis

The indirect ELISA data were analyzed using GraphPad Prism 9 (GraphPad Software Inc. USA) using paired t-tests, and the results are presented as mean ± SD. Differences between means were significant when p ≤ 0.05.

## Results

### Expression of p30 and SUMO-p30 in *E. coli*

**Figure-1:**
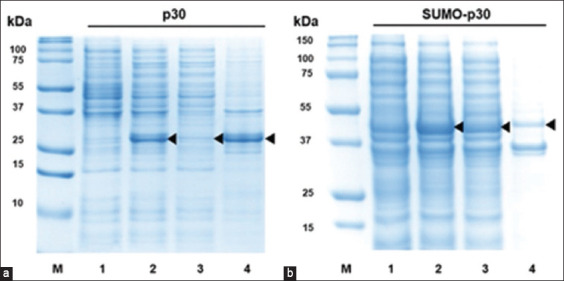
SDS-PAGE demonstrating the expression level and solubility of p30 (a) and SUMO-p30 (b) in *Escherichia coli* after IPTG induction. M: Molecular weight marker; 1: No IPTG induction of protein expression; 2: IPTG induced protein expression; 3: Protein in the soluble fraction; 4: Protein in the insoluble fraction. IPTG=Isopropyl β-D-thiogalactoside. SUMO=Small ubiquitin-like modifier, SDS-PAGE=Sodium dodecyl sulfate-polyacrylamide gel electrophoresis.

Both p30 and SUMO-p30 proteins were produced in *E*. *coli* strain BL21. SUMO-p30 and p30 without the SUMO-tag exhibited molecular weights of 45 and 30 kDa, respectively (Figure-1a). SDS-PAGE results indicated that the expression level of SUMO-p30 was higher than that of p30 alone. To compare protein solubility, we extracted p30 and SUMO-p30 in both the soluble and insoluble forms. We found that p30 was primarily found in the insoluble fraction, while the majority of SUMO-p30 was present in the soluble fraction (Figure-1b). These results confirmed that the SUMO-tag not only improved the solubility but also enhanced p30 expression in *E. coli*.

### Partial purification of p30 and SUMO-p30 proteins using ammonium sulfate

**Figure-2:**
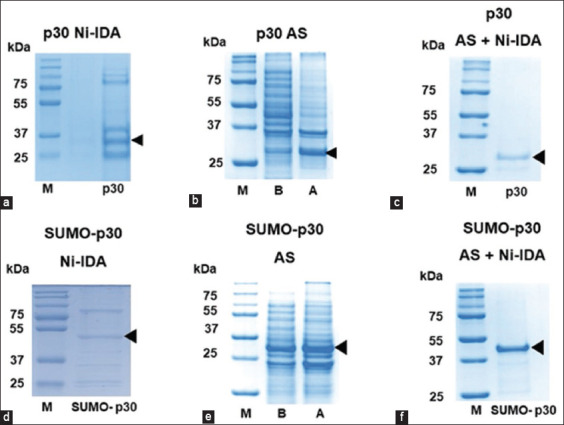
SDS-PAGE showing protein profiles of purified p30 and SUMO-p30. Qualities of p30 (a, b, and c) and SUMO-p30 (d, e, and f) partially purified by ammonium sulphate (AS) precipitation (b and e) followed by Ni-IDA affinity chromatography (c and f) can be observed to be higher than those purified with affinity chromatography alone (a and d). M: molecular weight marker. B: Before AS precipitation. A: After AS precipitation. SUMO=Small ubiquitin-like modifier, SDS-PAGE=Sodium dodecyl sulfate-polyacrylamide gel electrophoresis, IDA=Iminodiacetic acid.

Direct purification of the soluble forms of both p30 and SUMO-p30 by affinity chromatography using the Ni-IDA resin column was initially attempted. However, the expression of p30 in soluble form in *E. coli* was quite low, and both p30 and SUMO-p30 were heavily contaminated with host proteins. Consequently, purification by affinity chromatography alone could not effectively remove these contaminating proteins from p30 (Figure-2a and d). To improve the purity of p30, ammonium sulfate precipitation was used to remove non-specific proteins before affinity chromatography purification. Modified step-wise ammonium sulfate precipitation was performed to determine the optimal level of ammonium sulfate saturation to separate p30 and SUMO-p30 from the contaminated proteins (Table-1). Some contaminated proteins were precipitated at 10% ammonium sulfate saturation, while p30 was completely precipitated at 20% ammonium sulfate saturation. In the case of SUMO-p30 protein precipitation, it began to precipitate at 30% saturation and was completely precipitated at 60% ammonium sulfate saturation. The procedure consisted of two steps: The first step is the precleared step, in which some contaminant proteins are eliminated by precipitation. At this stage, p30 and SUMO-p30 remained dissolved at ammonium sulfate concentrations of 10% and 30%, respectively. The final step was the precipitation of the target protein, during which the ammonium sulfate content of the supernatants containing p30 or SUMO-p30 was further increased to 20% and 60%, respectively. In this step, the protein of interest was precipitated and the dissolved contaminant proteins were discarded. SDS-PAGE results revealed that p30 and SUMO-p30 clearly appeared after partial purification with ammonium sulfate precipitation (Figure-2b and e).

**Table 1:** Percentage optimization of saturated ammonium sulfate suitable for precipitating p30 and SUMO-p30.

Proteins	Percentage saturation of ammonium sulfate					

	10%	20%	30%	40%	50%	60%
p30	No precipitation	Complete precipitation	Complete precipitation	Complete precipitation	Complete precipitation	Complete precipitation
SUMO-p30	No precipitation	No precipitation	Partial precipitation	Partial precipitation	Partial precipitation	Complete precipitation

SUMO=Small ubiquitin-like modifier

### Purification of the p30 and SUMO-p30 proteins using affinity chromatography

After ammonium sulfate precipitation, p30 and SUMO-p30 were further purified by affinity chromatography on the basis of the binding between nickel ions and histidine tag fusion proteins. The p30 and SUMO-p30 were eluted using an elution buffer containing 100 mM imidazole. Prior partial purification with ammonium sulfate significantly increased the purity of both p30 and SUMO-p30. SDS-PAGE analysis demonstrated that the purity of both proteins exceeded 95% (Figures-2c and f) compared with that without ammonium sulfate precipitation (Figures-2a and d).

### Cleavage and removal of SUMO-tag from SUMO-p30 protein

**Figure-3:**
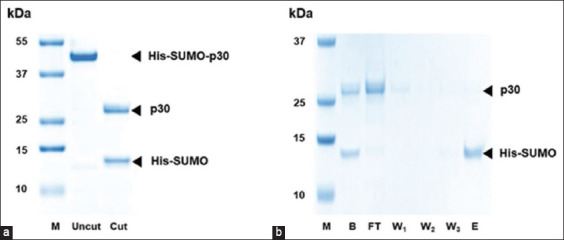
SDS-PAGE showing the results of enzymatic cleavage and removal of SUMO tag. SUMO-p30 cleaved with SUMO protease 1 (a). Removal of 6×His-SUMO tag, accomplished by reintroducing the protein solution into the Ni-IDA resin column (b). M: Molecular weight marker; Uncut: SUMO-p30; Cut: SUMO-cleaved p30; B: before removing 6ÍHis-SUMO tag, FT: SUMO-cleaved p30 in the flow-through fraction, W1-3: Washed fractions containing 20-, 50-, and 100-mM imidazole, respectively. E: Eluted fraction containing 250 mM imidazole. SUMO=Small ubiquitin-like modifier, SDS-PAGE=Sodium dodecyl sulfate-polyacrylamide gel electrophoresis, IDA=Iminodiacetic acid.

The SUMO-tag may interfere with the p30 property, so it was removed from the protein. Cleavage of the SUMO-tag from SUMO-p30 was achieved by treatment with SUMO protease 1 after incubation at 30°C for 1 h. After incubation with the enzyme, 30 kDa p30 and 12 kDa 6His-SUMO-tag fusion proteins were distinctly separated in the gel (Figure-3a). The cleaved 6His-SUMO-tag and protease were subsequently removed from the protein mixture by reintroducing the protein solutions into the Ni-IDA resin column. p30 appeared in the flow-through fraction, whereas the 6His-SUMO-tag and protease were retained in the column and subsequently released in the elution buffer (Figure-3b). The 6His-SUMO protease protein band was not observed since its concentration in the gel was lower than the detection limit of Coomassie Brilliant Blue G-250. Concentrations of purified p30, SUMO-p30, and SUMO-cleaved p30 were 1.02, 6.59, and 3.13 μg/mL, respectively.

### Examination of p30 immunogenicity

**Figure-4:**
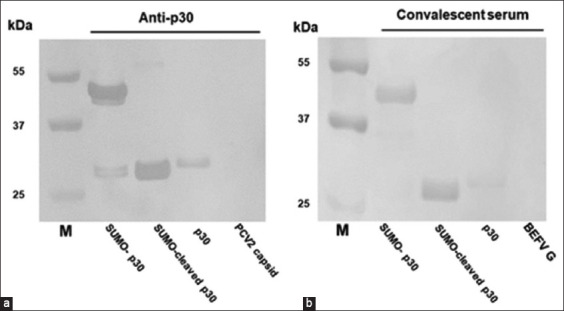
Western blots demonstrating the immunogenicity of p30 proteins. 5 μL of each protein loaded into the wells of polyacrylamide gels for electrophoresis. The gels are blotted onto nitrocellulose membranes before incubating with a monoclonal antibody specific to p30 (a) or a swine convalescent serum (b). All versions of p30 can be observed to react with a monoclonal antibody specific to p30 (a) and a swine convalescent serum (b), while the capsid protein of porcine circovirus type 2 (a) and glycoprotein G of bovine ephemeral fever virus (b) can be observed not to react. M: Molecular weight marker.

To examine the immunogenicity of p30 proteins, SDS-PAGE and western blotting were performed with 5 μL of each protein. The blotted proteins were detected using p30-specific monoclonal antibody and convalescent serum from a pig in an ASF-affected farm in Western Thailand. As shown in Figure-4a, p30, SUMO-p30, and SUMO-cleaved p30 exhibited specific reactivity with the anti-p30 monoclonal antibody. Similarly, they reacted with convalescent swine serum without any cross-reactivity observed under the negative antigen control (Figure-4b). These results confirmed that all versions of the recombinant proteins expressed from *E. coli* were ASFV p30.

### Large-scale expression and purification of p30 and SUMO-p30

**Figure-5:**
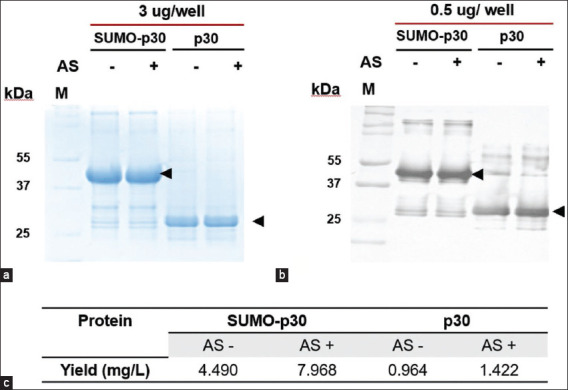
Comparison of p30 and SUMO-p30 with and without partial purification by ammonium sulfate precipitation. The images show protein profiles in SDS-PAGE (a), their immunogenicity in western blots (b), and the protein yields in milligrams per liter of *Escherichia coli* culture (c). M: Molecular weight marker. AS: Ammonium sulfate precipitation. SUMO=Small ubiquitin-like modifier, SDS-PAGE=Sodium dodecyl sulfate-polyacrylamide gel electrophoresis.

To enhance the protein production process, 1 L of each *E. coli* culture was prepared under four conditions: p30 and SUMO-p30 purified solely by affinity chromatography and p30 and SUMO-p30 with ammonium sulfate precipitation, followed by affinity chromatography. The prepacked HisTrap-HP column was exploited for affinity chromatography using the automated AKTA™ start chromatography system (Cytiva, Marlborough, Massachusetts, USA) to accommodate large-scale production. p30 and SUMO-p30 were eluted from the columns using elution buffer containing 100–500 mM imidazole. According to the SDS-PAGE results, the intensity of the protein bands in the highly pure fractions of both p30 and SUMO-p30 purified by ammonium sulfate precipitation combined with affinity chromatography was higher than that obtained by affinity chromatography alone. SDS-PAGE analysis showed that the purity of the pooled fractions of SUMO-p30 and p30 with and without partial purification were comparable (Figure-5a). Western blot analysis also showed that p30 strongly reacted with the convalescent swine serum, similar to that obtained from small-scale production (Figure-5b). The yields of p30 and SUMO-p30 with ammonium precipitation, followed by affinity chromatography, were 1.422 and 7.968 mg/L, respectively, and those of p30 and SUMO-p30 purified by affinity chromatography alone were 0.964 and 4.490 mg/L, respectively (Figure-5c).

### Application of p30 in an indirect ELISA

**Figure-6:**
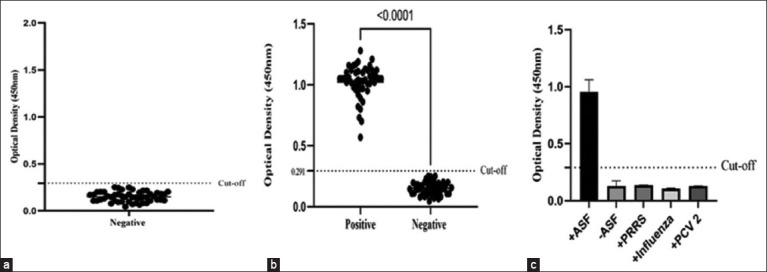
Performance of indirect ELISA based on SUMO-cleaved p30. The cutoff value of the p30 indirect ELISA (a) was 0.291 by setting at mean OD of negative sera plus three-fold standard deviations (X̅ + 3SD). Scatter plots of OD450 of swine serum samples obtained from the p30 indirect ELISA showing a significant difference in the OD450 of positive and negative serum samples (b). The p30 indirect ELISA does not react with serums raised against the porcine reproductive and respiratory syndrome virus, swine influenza virus, and porcine circovirus type 2 (c). SUMO=Small ubiquitin-like modifier, ELISA=Enzyme-linked immunosorbent assay, OD=Optical density.

Subsequently, we evaluated the potential use of SUMO-cleaved p30 as an antigen for ASFV detection. Checkerboard titration of SUMO-cleaved p30 and convalescent swine serum was performed to establish the indirect ELISA. The optimal concentration of the SUMO-cleaved p30 protein for coating each well was 0.025 μg (0.25 μg/mL), with a cutoff value of 0.291 at the mean of ASF-negative sera plus 3SD (Figure-6a). The p30 indirect ELISA performance was then evaluated using 128 positive and 72 negative pig serum samples previously determined by the ID Screen® ASF Indirect. The results showed that this assay could discriminate between positive and negative serum samples, with statistically significant differences in the mean OD450 values between positive and negative sera (p < 0.001) (Figure-6b). Inter- and intra-plate assays were also performed to evaluate the reproducibility of the assay. The results revealed low variations within and between plates, with CVs of <10% (Table-2). Subsequently, this study examined whether SUMO-cleaved p30 could cross-react with antibodies specific to other swine viral pathogens. p30 did not react with serum samples from PCV2, SIV, or PRRSV-infected pigs (Figure-6c).

**Table 2:** Repeatability of ELISA in intra- and inter-plate assays.

Serum sample No.	Intra-assay		Inter-assay	
	
	X̅ ± SD	CV (%)	X̅ ± SD	CV (%)
Positive				
1	1.476 ± 0.042	2.85	1.236 ± 0.016	1.32
2	1.462 ± 0.075	5.15	1.344 ± 0.017	1.23
3	1.330 ± 0.057	4.22	1.233 ± 0.020	1.62
4	1.329 ± 0.050	3.77	1.106 ± 0.013	1.18
Negative				
1	0.104 ± 0.004	3.72	0.072 ± 0.002	2.97
2	0.097 ± 0.003	2.73	0.066 ± 0.002	2.93
3	0.092 ± 0.003	2.96	0.068 ± 0.003	4.61
4	0.112 ± 0.002	1.42	0.087 ± 0.001	1.17

X̅=Mean, SD=Standard deviation; CV (%)=Coefficient of variation; ELISA=Enzyme-linked immunosorbent assay

## Discussion

*E. coli* as a host for recombinant protein production is a well-established choice due to its rapidity, cost-effectiveness, and ease of handling. However, protein aggregation and inclusion body formation can occur during *E. coli* expression [31]. At present, various fusion tags, including thioredoxin (Trx), glutathione-S-transferase, maltose-binding protein, and SUMO, have been used to improve the expression level and solubility of foreign proteins in *E. coli*. Numerous studies have demonstrated that SUMO enhances the expression and solubility of fused proteins [20, 32]. For example, SUMO significantly increases the expression level and improves the solubility of protein partners such as Kras4BG15V oncoprotein, OmpC of *Salmonella* Typhimurium, and Abaecin antimicrobial peptide [20], human urocortin 2 [32], and severe acute respiratory syndrome coronavirus proteins [24, 25]. Recently, PmoB subunit of particulate methane monooxygenase (pMMO) was successfully generated as a SUMO fusion protein in a cell-free expression system. The SUMO-tag was enzymatically cleaved to release the native structure of PmoB in the reaction assembled with PmoA and PmoC to form a complete pMMO complex [33].

Previous attempts to produce recombinant ASFV p30 for diagnostic purposes have often resulted in the formation of inclusion bodies requiring purification under denaturing conditions [12, 13, 34, 35]. This denaturation process may potentially affect the native structure and antigenicity of p30. Therefore, a SUMO fusion tag in combination with a histidine tag was selected in this study to enhance the solubility and purification feasibility of ASFV p30. The SUMO fusion tag significantly increased the expression levels and solubility of ASFV p30 in *E. coli*. Although the expression of recombinant p30 fused with the Trx tag in *E. coli* was in the native soluble form [11], the specificity of the SUMO-tag for cleavage by the cognate tag-cleaving protease may provide an advantage. In fact, protein tag removal may be necessary since fusion tags can potentially interfere with partner protein structure and function [21]. However, cleavage of the fusion tag may lead to protein precipitation and aggregation because the fusion tag plays a crucial role in maintaining the solubility of the partner proteins. The highly specific SUMO protease recognizes the tertiary structure of SUMO rather than the amino acid sequence [18]. Therefore, it does not obliterate the protein partner [16]. In addition, it cleaves at the C-terminus of the SUMO sequence, leaving no exogenous residues on the cleaved protein [23]. The SUMO-tag was cleaved without nonspecific proteolysis in this study. Separation of SUMO-tag from protein of interest is also practical when fused with the histidine tag. This study demonstrates that tag removal and separation from the soluble native ASFV p30 can be accomplished by incubating with SUMO protease and subsequently reintroducing the protein solution into the Ni-IDA resin column.

This study revealed significantly lower expression levels of soluble p30 without the SUMO-tag. Both p30 and SUMO-p30 were contaminated with non-specific proteins that could compete for binding to the Ni-affinity resin [36]. The problem was resolved by pre-clearing and removing contaminants using ammonium sulfate precipitation, based on the different solubility of proteins in solutions containing different concentrations of ammonium sulfate salt [37]. It is commonly used for partial purification of antibodies [38]. The method of ammonium sulfate precipitation in a stepwise manner has demonstrated significant efficacy in the removal of contaminant proteins. These contaminants previously prevented the binding of nickel ions to the histidine tag during affinity purification. Ammonium sulfate precipitation improved the purity and concentration of ASFV p30 before purification by Ni-IDA affinity chromatography. The yield of affinity-purified p30 was significantly lower than that of SUMO-cleaved p30. The increase in production can be attributed to the presence of the SUMO-tag.

To date, recombinant p30 has been produced using various expression systems, including mammalian cells, insect cells, and *E. coli*. Recombinant p30 production in mammalian and insect cells [39, 40] presents an advantage over *E. coli* hosts because the expressed protein undergoes post-translational modifications, including phosphorylation. Previous studies by Liberti and colleague [40] have demonstrated that insect cell-derived recombinant p30 exhibits high yield and purity, whereas p30 expressed in mammalian cells closely resembles the native protein [39]. However, foreign protein expression in *E. coli* hosts remains a demand due to the cost-effectiveness and less intricate production process [41].

Recombinant p30 produced in *E*. *coli* is widely employed in indirect ELISA [42], blocking ELISA [13], and immunoblotting assays [11] for diagnostic purposes. The recombinant p30 produced in this study exhibited a high level of solubility and strong reactivity with ASFV-positive sera in both western blot and indirect ELISA. These findings suggest the potential utility of p30 proteins for diagnostic and immunization purposes.

## Conclusion

In this study, ASFV p30 was cloned, fused in-frame with histidine and SUMO tags, and the proteins were expressed in *E. coli*. Incorporation of SUMO-tag significantly enhances the expression level and solubility of p30 protein, resulting in soluble native conformation of most of the protein. In addition, the quality of p30 is further enhanced by a dual purification process consisting of ammonium sulfate precipitation and affinity chromatography. In addition, the cleavage and separation of SUMO-tag from p30 have been proven to be a straightforward and convenient procedure, which makes it suitable for upscaling. Purified p30 also retains high antigenicity. Hence, p30 cloning, expression, and purification methods hold substantial promise for producing not only p30 but also other proteins.

## Authors’ Contributions

PL and PH: Conceptualization; JC, PH, PL, and NT: Methodology; JC: Data curation; JC, PH, and PL: Validation; JC, PH, NT, ST, and PC: Investigation; JC: Visualization; JC and PH: Writing-original draft preparation; PL: Writing-review and editing; PL and CK: Resources; PL, PH, NT, ST and CK: Supervision; PL and PH: Project administration. All authors have read, reviewed, and approved the manuscript.

## References

[1] Montgomery R.E (1921). On a form of swine fever occurring in British East Africa (Kenya Colony). J. Comp. Pathol. Therapeut.

[2] Gaudreault N.N, Madden D.W, Wilson W.C, Trujillo J.D, Richt J.A (2020). *African swine fever virus*:An emerging DNA arbovirus. Front. Vet. Sci.

[3] Dixon L.K, Stahl K, Jori F, Vial L, Pfeiffer D.U (2020). African swine fever epidemiology and control. Annu. Rev. Anim. Biosci.

[4] Ge S, Li J, Fan X, Liu F, Li L, Wang Q, Ren W, Bao J, Liu C, Wang H, Liu Y, Zhang Y, Xu T, Wu X, Wang Z (2018). Molecular characterization of *African swine fever virus*, China. Emerg. Infect. Dis.

[5] Mighell E, Ward M.P (2021). African swine fever spread across Asia, 2018–2019. Transbound. Emerg. Dis.

[6] You S, Liu T, Zhang M, Zhao X, Dong Y, Wu B, Wang Y, Li J, Wei X, Shi B (2021). African swine fever outbreaks in China led to gross domestic product and economic losses. Nat. Food.

[7] Alonso C, Borca M, Dixon L, Revilla Y, Rodriguez F, Escribano J.M, ICTV Report Consortium (2018). ICTV virus taxonomy profile:*Asfarviridae*. J. Gen. Virol.

[8] Alejo A, Matamoros T, Guerra M, Andrés G (2018). A proteomic atlas of the *African swine fever virus* particle. J. Virol.

[9] Cubillos C, Gómez-Sebastian S, Moreno N, Nuñez M.C, Mulumba-Mfumu L.K, Quembo C.J, Heath L, Etter E.M, Jori F, Escribano J.M, Blanco E (2013). *African swine fever virus* serodiagnosis:A general review with a focus on the analyses of African serum samples. Virus Res.

[10] Gómez-Puertas P, Rodríguez F, Oviedo J.M, Brun A, Alonso C, Escribano J.M (1998). The *African swine fever virus* proteins p54 and p30 are involved in two distinct steps of virus attachment and both contribute to the antibody-mediated protective immune response. Virology.

[11] Kazakova A.S, Imatdinov I.R, Dubrovskaya O.A, Imatdinov A.R, Sidlik M.V, Balyshev V.M, Krasochko P.A, Sereda A.D (2017). Recombinant protein p30 for serological diagnosis of African swine fever by immunoblotting assay. Transbound. Emerg. Dis.

[12] Giménez-Lirola L.G, Mur L, Rivera B, Mogler M, Sun Y, Lizano S, Goodell C, Harris D.L, Rowland R.R, Gallardo C, Sánchez-Vizcaíno J.M, Zimmerman J (2016). Detection of *African swine fever virus* antibodies in serum and oral fluid specimens using a recombinant protein 30 (p30) dual matrix indirect ELISA. PLoS One.

[13] Yu X, Zhu X, Chen X, Li D, Xu Q, Yao L, Sun Q, Ghonaim A.H, Ku X, Fan S, Yang H, He Q (2021). Establishment of a blocking ELISA detection method for against *African swine fever virus* p30 antibody. Front. Vet. Sci.

[14] Rosano G.L, Ceccarelli E.A (2014). Recombinant protein expression in *Escherichia coli*:Advances and challenges. Front. Microbiol.

[15] Hannoun Z, Greenhough S, Jaffray E, Hay R.T, Hay D.C (2010). Post-translational modification by SUMO. Toxicology.

[16] Butt T.R, Edavettal S.C, Hall J.P, Mattern M.R (2005). SUMO fusion technology for difficult-to-express proteins. Protein Expr. Purif.

[17] Marblestone J.G, Edavettal S.C, Lim Y, Lim P, Zuo X, Butt T.R (2006). Comparison of SUMO fusion technology with traditional gene fusion systems:Enhanced expression and solubility with SUMO. Protein Sci.

[18] Malakhov M.P, Mattern M.R, Malakhova O.A, Drinker M, Weeks S.D, Butt T.R (2004). SUMO fusions and SUMO-specific protease for efficient expression and purification of proteins. J. Struct. Funct. Genomics.

[19] Kim D.S, Kim S.W, Song J.M, Kim S.Y, Kwon K.C (2019). A new prokaryotic expression vector for the expression of antimicrobial peptide abaecin using SUMO fusion tag. BMC Biotechnol.

[20] Prejit Pratheesh, P.T Nimisha, S Jess, V Asha, K. and Agarwal R.K (2019). Expression and purification of an immunogenic SUMO-OmpC fusion protein of *Salmonella Typhimurium* in *Escherichia coli*. Biologicals.

[21] Park A.R, Kim S.W, Kim S.Y, Kwon K.C (2021). Expression of antimicrobial peptide (AMP), Cecropin B, in a fused form to SUMO tag with or without three-glycine linker in *Escherichia coli* and evaluation of bacteriolytic activity of the purified AMP. Probiotics Antimicrob.

[22] Wu Y, Hua H, Huang Z, Feng M, Feng J (2020). Cloning, expression, and purification of porcine adrenocorticotropic hormone in *Escherichia coli*. Protein Expr. Purif.

[23] Munir A, Ahmed N, Akram M, Fujimura N.A, Tahir S, Malik K (2023). Enhanced soluble expression of active recombinant human interleukin-29 using champion pET SUMO system. Biotechnol. Lett.

[24] Zuo X, Mattern M.R, Tan R, Li S, Hall J, Sterner D.E, Shoo J, Tran H, Lim P, Sarafianos S.G, Kazi L, Navas-Martin S, Weiss S.R, Butt T.R (2005). Expression and purification of SARS coronavirus proteins using SUMO-fusions. Protein Expr. Purif.

[25] Zuo X, Li S, Hall J, Mattern M.R, Tran H, Shoo J, Tan R, Weiss S.R, Butt T.R (2005). Enhanced expression and purification of membrane proteins by SUMO fusion in *Escherichia coli*. J. Struct. Funct. Genomics.

[26] Sariya L, Thangthumniyom N, Wajjwalku W, Chumsing W, Ramasoota P, Lekcharoensuk P (2011). Expression of foot and mouth disease virus nonstructural polyprotein 3ABC with inactive 3C(pro) in *Escherichia coli*. Protein Expr. Purif.

[27] Burgess R.R (2009). Protein precipitation techniques. Methods Enzymol.

[28] Hansoongnern P, Kaewborisuth C, Wasanasuk K, Chankeeree P, Poonsuk S, Lekcharoensuk C, Lekcharoensuk P (2019). The immunogenicity of the secretory GΔTM protein of bovine ephemeral fever virus stably expressed by mammalian cells. Vet. Microbiol.

[29] Hansoongnern P, Phecharat N, Wasanasuk K, Tommeurd W, Chankeeree P, Lekcharoensuk C, Semkum P, Pinitkiatisakul S, &Lekcharoensuk P (2022). Encapsidated-CpG ODN enhances immunogenicity of porcine circovirus type 2 virus-like particles. Vet. Microbiol.

[30] Srisombundit V, Tungthumniyom N, Linchongsubongkoch W, Lekcharoensuk C, Sariya L, Ramasoota P, Lekcharoensuk P (2013). Development of an inactivated 3Cpro-3ABC (mu3ABC) ELISA to differentiate cattle infected with foot and mouth disease virus from vaccinated cattle. J. Virol. Methods.

[31] Kaur J, Kumar A, &Kaur J (2018). Strategies for optimization of heterologous protein expression in *E. coli*:Roadblocks and reinforcements. Int. J. Biol. Macromol.

[32] Liew O.W, Ang C.X, Peh Y.P, Chong P.C, Ng Y.X, Hwang L.A, Koh X.Y, Yip Y.M, Liu W, Richards A.M (2014). A His6-SUMO-eXact tag for producing human prepro-urocortin 2 in *Escherichia coli* for raising monoclonal antibodies. J. Immunol. Methods.

[33] Koo C. W, Hershewe J. M, Jewett M. C, Rosenzweig A. C (2022). Cell-free protein synthesis of particulate methane monooxygenase into nanodiscs. ACS Synth. Biol.

[34] Zhou J, Ni Y, Wang D, Fan B, Zhu X, Zhou J, Hu Y, Li L, Li B (2023). Development of a competitive enzyme-linked immunosorbent assay targeting the-p30 protein for detection of antibodies against African swine fever virus. Viruses.

[35] Yuan F, Petrovan V, Gimenez-Lirola L. G, Zimmerman J. J, Rowland R. R. R, Fang Y (2021). Development of a blocking enzyme-linked immunosorbent assay for detection of antibodies against African swine fever virus. Pathogens (Basel, Switzerland).

[36] Bornhorst J. A, Falke J. J (2000). Purification of proteins using polyhistidine affinity tags. Methods Enzymol.

[37] Wingfield P (2001). Protein precipitation using ammonium sulfate. Curr. Protoc. Protein Sci.

[38] Fishman J. B, Berg E. A (2018). Ammonium sulfate fractionation of antibodies. Cold Spring Harb. Protoc.

[39] Chen X, Chen X, Liang Y, Xu S, Weng Z, Gao Q, Huang Z, Zhang G, Gong L (2022). Interaction network of African swine fever virusstructural protein p30 with host proteins. Front. Microbiol.

[40] Liberti R, Colabella C, Anzalone L, Severi G, Di Paolo A, Casciari C, Casano A. B, Giammarioli M, Cagiola M, Feliziani F, De Giuseppe A (2023). Expression of a recombinant ASFV P30 protein and production of monoclonal antibodies. Open Vet. J.

[41] Arya R, Bhattacharya A, Saini K. S (2008). Dictyostelium discoideum--a promising expression system for the production of eukaryotic proteins. FASEB J.

[42] Li D, Zhang Q, Liu Y, Wang M, Zhang L, Han L, Chu X, Ding G, Li Y, Hou Y, Liu S, Wang Z, Xiao Y (2022). Indirect ELISA Using multi-antigenic dominants of p30, p54 and p72 recombinant proteins to detect antibodies against African swine fever virus in pigs. Viruses.

